# Supplementary material for: Pyruvate Dehydrogenase Kinase 1 inhibition mediated oxidative phosphorylation enhancement in cartilage promotes osteoarthritis progression
Source: BMC Musculoskelet Disord. 2023 Jul 20;24:597. doi: 10.1186/s12891-023-06585-6 (PMC10357736; doi:10.1186/s12891-023-06585-6)
Supplement: Supplementary file 1 — Additional file 1. [file 12891_2023_6585_MOESM1_ESM.zip › BP.Gsea.1653623667859/GOBP_PURINE_CONTAINING_COMPOUND_BIOSYNTHETIC_PROCESS.html]

Details for gene set GOBP\_PURINE\_CONTAINING\_COMPOUND\_BIOSYNTHETIC\_PROCESS[GSEA]

|  || Dataset | 55457\_collapsed\_to\_symbols.cls.cls #OA\_versus\_normal.cls.cls #OA\_versus\_normal\_repos |
| Phenotype | cls.cls#OA\_versus\_normal\_repos |
| Upregulated in class | OA |
| GeneSet | GOBP\_PURINE\_CONTAINING\_COMPOUND\_BIOSYNTHETIC\_PROCESS |
| Enrichment Score (ES) | 0.28642222 |
| Normalized Enrichment Score (NES) | 1.4516225 |
| Nominal p-value | 0.006688963 |
| FDR q-value | 0.42046475 |
| FWER p-Value | 1.0 |
Table: GSEA Results Summary

  

Fig 1: Enrichment plot: GOBP\_PURINE\_CONTAINING\_COMPOUND\_BIOSYNTHETIC\_PROCESS      
 Profile of the Running ES Score & Positions of GeneSet Members on the Rank Ordered List

  

| SYMBOL | TITLE | RANK IN GENE LIST | RANK METRIC SCORE | RUNNING ES | CORE ENRICHMENT || 1 | DGUOK | deoxyguanosine kinase [Source:HGNC Symbol;Acc:HGNC:2858] | 96 | 0.917 | 0.0083 | Yes |
| 2 | ADK | adenosine kinase [Source:HGNC Symbol;Acc:HGNC:257] | 127 | 0.878 | 0.0212 | Yes |
| 3 | MPC2 | mitochondrial pyruvate carrier 2 [Source:HGNC Symbol;Acc:HGNC:24515] | 140 | 0.870 | 0.0353 | Yes |
| 4 | PDHB | pyruvate dehydrogenase E1 subunit beta [Source:HGNC Symbol;Acc:HGNC:8808] | 149 | 0.862 | 0.0496 | Yes |
| 5 | NME7 | NME/NM23 family member 7 [Source:HGNC Symbol;Acc:HGNC:20461] | 178 | 0.835 | 0.0619 | Yes |
| 6 | PANK2 | pantothenate kinase 2 [Source:HGNC Symbol;Acc:HGNC:15894] | 220 | 0.805 | 0.0726 | Yes |
| 7 | GUCA1A | guanylate cyclase activator 1A [Source:HGNC Symbol;Acc:HGNC:4678] | 316 | 0.739 | 0.0779 | Yes |
| 8 | TREM2 | triggering receptor expressed on myeloid cells 2 [Source:HGNC Symbol;Acc:HGNC:17761] | 342 | 0.726 | 0.0885 | Yes |
| 9 | NME5 | NME/NM23 family member 5 [Source:HGNC Symbol;Acc:HGNC:7853] | 362 | 0.720 | 0.0995 | Yes |
| 10 | ATIC | 5-aminoimidazole-4-carboxamide ribonucleotide formyltransferase/IMP cyclohydrolase [Source:HGNC Symbol;Acc:HGNC:794] | 401 | 0.706 | 0.1087 | Yes |
| 11 | ATP5MC1 | ATP synthase membrane subunit c locus 1 [Source:HGNC Symbol;Acc:HGNC:841] | 465 | 0.674 | 0.1155 | Yes |
| 12 | AK1 | adenylate kinase 1 [Source:HGNC Symbol;Acc:HGNC:361] | 481 | 0.667 | 0.1258 | Yes |
| 13 | ATP5PD | ATP synthase peripheral stalk subunit d [Source:HGNC Symbol;Acc:HGNC:845] | 564 | 0.635 | 0.1304 | Yes |
| 14 | DLD | dihydrolipoamide dehydrogenase [Source:HGNC Symbol;Acc:HGNC:2898] | 565 | 0.635 | 0.1414 | Yes |
| 15 | PAPSS2 | 3'-phosphoadenosine 5'-phosphosulfate synthase 2 [Source:HGNC Symbol;Acc:HGNC:8604] | 592 | 0.627 | 0.1502 | Yes |
| 16 | ANTKMT | adenine nucleotide translocase lysine methyltransferase [Source:HGNC Symbol;Acc:HGNC:14152] | 618 | 0.618 | 0.1589 | Yes |
| 17 | NME4 | NME/NM23 nucleoside diphosphate kinase 4 [Source:HGNC Symbol;Acc:HGNC:7852] | 677 | 0.599 | 0.1647 | Yes |
| 18 | DMAC2L | distal membrane arm assembly component 2 like [Source:HGNC Symbol;Acc:HGNC:18799] | 695 | 0.595 | 0.1737 | Yes |
| 19 | PAPSS1 | 3'-phosphoadenosine 5'-phosphosulfate synthase 1 [Source:HGNC Symbol;Acc:HGNC:8603] | 703 | 0.593 | 0.1834 | Yes |
| 20 | PPCS | phosphopantothenoylcysteine synthetase [Source:HGNC Symbol;Acc:HGNC:25686] | 716 | 0.589 | 0.1927 | Yes |
| 21 | HSD17B12 | hydroxysteroid 17-beta dehydrogenase 12 [Source:HGNC Symbol;Acc:HGNC:18646] | 795 | 0.570 | 0.1964 | Yes |
| 22 | ATP5PO | ATP synthase peripheral stalk subunit OSCP [Source:HGNC Symbol;Acc:HGNC:850] | 840 | 0.558 | 0.2026 | Yes |
| 23 | NME3 | NME/NM23 nucleoside diphosphate kinase 3 [Source:HGNC Symbol;Acc:HGNC:7851] | 876 | 0.549 | 0.2094 | Yes |
| 24 | DCK | deoxycytidine kinase [Source:HGNC Symbol;Acc:HGNC:2704] | 920 | 0.537 | 0.2153 | Yes |
| 25 | ACOT7 | acyl-CoA thioesterase 7 [Source:HGNC Symbol;Acc:HGNC:24157] | 971 | 0.524 | 0.2205 | Yes |
| 26 | ACAT1 | acetyl-CoA acetyltransferase 1 [Source:HGNC Symbol;Acc:HGNC:93] | 1017 | 0.513 | 0.2258 | Yes |
| 27 | PPT1 | palmitoyl-protein thioesterase 1 [Source:HGNC Symbol;Acc:HGNC:9325] | 1042 | 0.508 | 0.2327 | Yes |
| 28 | TECR | "trans-2,3-enoyl-CoA reductase [Source:HGNC Symbol;Acc:HGNC:4551]" | 1047 | 0.508 | 0.2412 | Yes |
| 29 | APRT | adenine phosphoribosyltransferase [Source:HGNC Symbol;Acc:HGNC:626] | 1178 | 0.481 | 0.2393 | Yes |
| 30 | ATP5PB | ATP synthase peripheral stalk-membrane subunit b [Source:HGNC Symbol;Acc:HGNC:840] | 1293 | 0.459 | 0.2383 | Yes |
| 31 | ATP5MF | ATP synthase membrane subunit f [Source:HGNC Symbol;Acc:HGNC:848] | 1330 | 0.453 | 0.2434 | Yes |
| 32 | ADCY7 | adenylate cyclase 7 [Source:HGNC Symbol;Acc:HGNC:238] | 1408 | 0.442 | 0.2450 | Yes |
| 33 | NME2 | NME/NM23 nucleoside diphosphate kinase 2 [Source:HGNC Symbol;Acc:HGNC:7850] | 1428 | 0.439 | 0.2511 | Yes |
| 34 | SHMT2 | serine hydroxymethyltransferase 2 [Source:HGNC Symbol;Acc:HGNC:10852] | 1518 | 0.426 | 0.2515 | Yes |
| 35 | ADCY1 | adenylate cyclase 1 [Source:HGNC Symbol;Acc:HGNC:232] | 1589 | 0.415 | 0.2531 | Yes |
| 36 | GUK1 | guanylate kinase 1 [Source:HGNC Symbol;Acc:HGNC:4693] | 1640 | 0.407 | 0.2563 | Yes |
| 37 | PDK2 | pyruvate dehydrogenase kinase 2 [Source:HGNC Symbol;Acc:HGNC:8810] | 1772 | 0.388 | 0.2527 | Yes |
| 38 | AMPD1 | adenosine monophosphate deaminase 1 [Source:HGNC Symbol;Acc:HGNC:468] | 1789 | 0.385 | 0.2581 | Yes |
| 39 | SNCA | synuclein alpha [Source:HGNC Symbol;Acc:HGNC:11138] | 1800 | 0.384 | 0.2640 | Yes |
| 40 | STOML2 | stomatin like 2 [Source:HGNC Symbol;Acc:HGNC:14559] | 1819 | 0.382 | 0.2692 | Yes |
| 41 | ADA2 | adenosine deaminase 2 [Source:HGNC Symbol;Acc:HGNC:1839] | 1955 | 0.367 | 0.2649 | Yes |
| 42 | ELOVL4 | ELOVL fatty acid elongase 4 [Source:HGNC Symbol;Acc:HGNC:14415] | 2017 | 0.360 | 0.2664 | Yes |
| 43 | PINK1 | PTEN induced kinase 1 [Source:HGNC Symbol;Acc:HGNC:14581] | 2097 | 0.350 | 0.2662 | Yes |
| 44 | PRPSAP1 | phosphoribosyl pyrophosphate synthetase associated protein 1 [Source:HGNC Symbol;Acc:HGNC:9466] | 2112 | 0.348 | 0.2711 | Yes |
| 45 | PDK1 | pyruvate dehydrogenase kinase 1 [Source:HGNC Symbol;Acc:HGNC:8809] | 2124 | 0.347 | 0.2763 | Yes |
| 46 | ATP5F1C | ATP synthase F1 subunit gamma [Source:HGNC Symbol;Acc:HGNC:833] | 2190 | 0.339 | 0.2771 | Yes |
| 47 | PPCDC | phosphopantothenoylcysteine decarboxylase [Source:HGNC Symbol;Acc:HGNC:28107] | 2251 | 0.332 | 0.2781 | Yes |
| 48 | ATP5MC2 | ATP synthase membrane subunit c locus 2 [Source:HGNC Symbol;Acc:HGNC:842] | 2371 | 0.318 | 0.2742 | Yes |
| 49 | PDHA1 | pyruvate dehydrogenase E1 subunit alpha 1 [Source:HGNC Symbol;Acc:HGNC:8806] | 2433 | 0.310 | 0.2748 | Yes |
| 50 | SPHK2 | sphingosine kinase 2 [Source:HGNC Symbol;Acc:HGNC:18859] | 2478 | 0.305 | 0.2766 | Yes |
| 51 | TGFB1 | transforming growth factor beta 1 [Source:HGNC Symbol;Acc:HGNC:11766] | 2490 | 0.303 | 0.2810 | Yes |
| 52 | ACSBG1 | acyl-CoA synthetase bubblegum family member 1 [Source:HGNC Symbol;Acc:HGNC:29567] | 2520 | 0.301 | 0.2840 | Yes |
| 53 | ATP5MC3 | ATP synthase membrane subunit c locus 3 [Source:HGNC Symbol;Acc:HGNC:843] | 2643 | 0.286 | 0.2793 | Yes |
| 54 | ATP5F1D | ATP synthase F1 subunit delta [Source:HGNC Symbol;Acc:HGNC:837] | 2701 | 0.279 | 0.2797 | Yes |
| 55 | ATP6V0C | ATPase H+ transporting V0 subunit c [Source:HGNC Symbol;Acc:HGNC:855] | 2790 | 0.271 | 0.2775 | Yes |
| 56 | GUCY2D | "guanylate cyclase 2D, retinal [Source:HGNC Symbol;Acc:HGNC:4689]" | 2832 | 0.267 | 0.2789 | Yes |
| 57 | PRPSAP2 | phosphoribosyl pyrophosphate synthetase associated protein 2 [Source:HGNC Symbol;Acc:HGNC:9467] | 2855 | 0.265 | 0.2817 | Yes |
| 58 | IMPDH2 | inosine monophosphate dehydrogenase 2 [Source:HGNC Symbol;Acc:HGNC:6053] | 2867 | 0.264 | 0.2854 | Yes |
| 59 | ATP5MG | ATP synthase membrane subunit g [Source:HGNC Symbol;Acc:HGNC:14247] | 2941 | 0.256 | 0.2841 | Yes |
| 60 | GUCY2C | guanylate cyclase 2C [Source:HGNC Symbol;Acc:HGNC:4688] | 2982 | 0.252 | 0.2854 | Yes |
| 61 | ATP5F1B | ATP synthase F1 subunit beta [Source:HGNC Symbol;Acc:HGNC:830] | 3024 | 0.247 | 0.2864 | Yes |
| 62 | ELOVL6 | ELOVL fatty acid elongase 6 [Source:HGNC Symbol;Acc:HGNC:15829] | 3253 | 0.226 | 0.2724 | No |
| 63 | NPPC | natriuretic peptide C [Source:HGNC Symbol;Acc:HGNC:7941] | 3276 | 0.223 | 0.2745 | No |
| 64 | PID1 | phosphotyrosine interaction domain containing 1 [Source:HGNC Symbol;Acc:HGNC:26084] | 3280 | 0.222 | 0.2782 | No |
| 65 | MTAP | methylthioadenosine phosphorylase [Source:HGNC Symbol;Acc:HGNC:7413] | 3324 | 0.219 | 0.2786 | No |
| 66 | PARP1 | poly(ADP-ribose) polymerase 1 [Source:HGNC Symbol;Acc:HGNC:270] | 3410 | 0.210 | 0.2755 | No |
| 67 | COASY | Coenzyme A synthase [Source:HGNC Symbol;Acc:HGNC:29932] | 3498 | 0.202 | 0.2722 | No |
| 68 | NT5E | 5'-nucleotidase ecto [Source:HGNC Symbol;Acc:HGNC:8021] | 3515 | 0.201 | 0.2744 | No |
| 69 | VCP | valosin containing protein [Source:HGNC Symbol;Acc:HGNC:12666] | 3518 | 0.201 | 0.2777 | No |
| 70 | PRPS1 | phosphoribosyl pyrophosphate synthetase 1 [Source:HGNC Symbol;Acc:HGNC:9462] | 3717 | 0.184 | 0.2654 | No |
| 71 | AK5 | adenylate kinase 5 [Source:HGNC Symbol;Acc:HGNC:365] | 3841 | 0.172 | 0.2587 | No |
| 72 | GUCY1B1 | guanylate cyclase 1 soluble subunit beta 1 [Source:HGNC Symbol;Acc:HGNC:4687] | 3962 | 0.159 | 0.2520 | No |
| 73 | ATP5F1A | ATP synthase F1 subunit alpha [Source:HGNC Symbol;Acc:HGNC:823] | 4137 | 0.143 | 0.2408 | No |
| 74 | AMPD3 | adenosine monophosphate deaminase 3 [Source:HGNC Symbol;Acc:HGNC:470] | 4174 | 0.140 | 0.2404 | No |
| 75 | AK2 | adenylate kinase 2 [Source:HGNC Symbol;Acc:HGNC:362] | 4179 | 0.140 | 0.2425 | No |
| 76 | NOS1 | nitric oxide synthase 1 [Source:HGNC Symbol;Acc:HGNC:7872] | 4228 | 0.134 | 0.2411 | No |
| 77 | NME6 | NME/NM23 nucleoside diphosphate kinase 6 [Source:HGNC Symbol;Acc:HGNC:20567] | 4257 | 0.132 | 0.2412 | No |
| 78 | ALDOA | "aldolase, fructose-bisphosphate A [Source:HGNC Symbol;Acc:HGNC:414]" | 4289 | 0.129 | 0.2410 | No |
| 79 | PRKAG2 | protein kinase AMP-activated non-catalytic subunit gamma 2 [Source:HGNC Symbol;Acc:HGNC:9386] | 4362 | 0.123 | 0.2374 | No |
| 80 | PRPS2 | phosphoribosyl pyrophosphate synthetase 2 [Source:HGNC Symbol;Acc:HGNC:9465] | 4365 | 0.123 | 0.2394 | No |
| 81 | GMPS | guanine monophosphate synthase [Source:HGNC Symbol;Acc:HGNC:4378] | 4498 | 0.113 | 0.2310 | No |
| 82 | NUDT2 | nudix hydrolase 2 [Source:HGNC Symbol;Acc:HGNC:8049] | 4521 | 0.112 | 0.2312 | No |
| 83 | MLYCD | malonyl-CoA decarboxylase [Source:HGNC Symbol;Acc:HGNC:7150] | 4852 | 0.083 | 0.2067 | No |
| 84 | ADCY9 | adenylate cyclase 9 [Source:HGNC Symbol;Acc:HGNC:240] | 5191 | 0.057 | 0.1812 | No |
| 85 | HACD1 | 3-hydroxyacyl-CoA dehydratase 1 [Source:HGNC Symbol;Acc:HGNC:9639] | 5342 | 0.044 | 0.1702 | No |
| 86 | PANK4 | pantothenate kinase 4 (inactive) [Source:HGNC Symbol;Acc:HGNC:19366] | 5382 | 0.041 | 0.1678 | No |
| 87 | SLC25A12 | solute carrier family 25 member 12 [Source:HGNC Symbol;Acc:HGNC:10982] | 5506 | 0.032 | 0.1587 | No |
| 88 | DLAT | dihydrolipoamide S-acetyltransferase [Source:HGNC Symbol;Acc:HGNC:2896] | 5647 | 0.020 | 0.1480 | No |
| 89 | ADSL | adenylosuccinate lyase [Source:HGNC Symbol;Acc:HGNC:291] | 5685 | 0.017 | 0.1454 | No |
| 90 | ACSBG2 | acyl-CoA synthetase bubblegum family member 2 [Source:HGNC Symbol;Acc:HGNC:24174] | 5970 | -0.002 | 0.1232 | No |
| 91 | ATP5PF | ATP synthase peripheral stalk subunit F6 [Source:HGNC Symbol;Acc:HGNC:847] | 6109 | -0.013 | 0.1126 | No |
| 92 | HACD2 | 3-hydroxyacyl-CoA dehydratase 2 [Source:HGNC Symbol;Acc:HGNC:9640] | 6162 | -0.017 | 0.1088 | No |
| 93 | NME1 | NME/NM23 nucleoside diphosphate kinase 1 [Source:HGNC Symbol;Acc:HGNC:7849] | 6328 | -0.031 | 0.0963 | No |
| 94 | ATP5ME | ATP synthase membrane subunit e [Source:HGNC Symbol;Acc:HGNC:846] | 6365 | -0.032 | 0.0941 | No |
| 95 | ATP5F1E | ATP synthase F1 subunit epsilon [Source:HGNC Symbol;Acc:HGNC:838] | 6405 | -0.036 | 0.0916 | No |
| 96 | ACSL6 | acyl-CoA synthetase long chain family member 6 [Source:HGNC Symbol;Acc:HGNC:16496] | 6505 | -0.043 | 0.0846 | No |
| 97 | SLC25A1 | solute carrier family 25 member 1 [Source:HGNC Symbol;Acc:HGNC:10979] | 6509 | -0.043 | 0.0851 | No |
| 98 | GUCY2F | "guanylate cyclase 2F, retinal [Source:HGNC Symbol;Acc:HGNC:4691]" | 6510 | -0.043 | 0.0859 | No |
| 99 | ADCY8 | adenylate cyclase 8 [Source:HGNC Symbol;Acc:HGNC:239] | 6646 | -0.053 | 0.0762 | No |
| 100 | IL4 | interleukin 4 [Source:HGNC Symbol;Acc:HGNC:6014] | 6673 | -0.054 | 0.0750 | No |
| 101 | GUCY1A2 | guanylate cyclase 1 soluble subunit alpha 2 [Source:HGNC Symbol;Acc:HGNC:4684] | 6731 | -0.058 | 0.0716 | No |
| 102 | PDHX | pyruvate dehydrogenase complex component X [Source:HGNC Symbol;Acc:HGNC:21350] | 6760 | -0.060 | 0.0704 | No |
| 103 | NPR2 | natriuretic peptide receptor 2 [Source:HGNC Symbol;Acc:HGNC:7944] | 6771 | -0.061 | 0.0707 | No |
| 104 | HPRT1 | hypoxanthine phosphoribosyltransferase 1 [Source:HGNC Symbol;Acc:HGNC:5157] | 6954 | -0.076 | 0.0577 | No |
| 105 | ADCY10 | adenylate cyclase 10 [Source:HGNC Symbol;Acc:HGNC:21285] | 7096 | -0.087 | 0.0481 | No |
| 106 | LDHC | lactate dehydrogenase C [Source:HGNC Symbol;Acc:HGNC:6544] | 7603 | -0.127 | 0.0106 | No |
| 107 | SLC26A1 | solute carrier family 26 member 1 [Source:HGNC Symbol;Acc:HGNC:10993] | 7618 | -0.128 | 0.0117 | No |
| 108 | PDK3 | pyruvate dehydrogenase kinase 3 [Source:HGNC Symbol;Acc:HGNC:8811] | 7661 | -0.132 | 0.0107 | No |
| 109 | MTHFD2L | methylenetetrahydrofolate dehydrogenase (NADP+ dependent) 2 like [Source:HGNC Symbol;Acc:HGNC:31865] | 7789 | -0.141 | 0.0032 | No |
| 110 | ATP6V1A | ATPase H+ transporting V1 subunit A [Source:HGNC Symbol;Acc:HGNC:851] | 8013 | -0.156 | -0.0116 | No |
| 111 | ELOVL2 | ELOVL fatty acid elongase 2 [Source:HGNC Symbol;Acc:HGNC:14416] | 8080 | -0.161 | -0.0140 | No |
| 112 | ADA | adenosine deaminase [Source:HGNC Symbol;Acc:HGNC:186] | 8105 | -0.163 | -0.0131 | No |
| 113 | ACSL3 | acyl-CoA synthetase long chain family member 3 [Source:HGNC Symbol;Acc:HGNC:3570] | 8170 | -0.169 | -0.0152 | No |
| 114 | ACLY | ATP citrate lyase [Source:HGNC Symbol;Acc:HGNC:115] | 8173 | -0.169 | -0.0124 | No |
| 115 | GCDH | glutaryl-CoA dehydrogenase [Source:HGNC Symbol;Acc:HGNC:4189] | 8180 | -0.170 | -0.0100 | No |
| 116 | PRPS1L1 | phosphoribosyl pyrophosphate synthetase 1 like 1 [Source:HGNC Symbol;Acc:HGNC:9463] | 8382 | -0.184 | -0.0226 | No |
| 117 | ELOVL1 | ELOVL fatty acid elongase 1 [Source:HGNC Symbol;Acc:HGNC:14418] | 8406 | -0.186 | -0.0211 | No |
| 118 | ADCY3 | adenylate cyclase 3 [Source:HGNC Symbol;Acc:HGNC:234] | 8572 | -0.199 | -0.0307 | No |
| 119 | SLC25A13 | solute carrier family 25 member 13 [Source:HGNC Symbol;Acc:HGNC:10983] | 8713 | -0.210 | -0.0380 | No |
| 120 | FLCN | folliculin [Source:HGNC Symbol;Acc:HGNC:27310] | 8848 | -0.221 | -0.0447 | No |
| 121 | PDHA2 | pyruvate dehydrogenase E1 subunit alpha 2 [Source:HGNC Symbol;Acc:HGNC:8807] | 9124 | -0.241 | -0.0621 | No |
| 122 | ACSL5 | acyl-CoA synthetase long chain family member 5 [Source:HGNC Symbol;Acc:HGNC:16526] | 9136 | -0.243 | -0.0588 | No |
| 123 | ACACA | acetyl-CoA carboxylase alpha [Source:HGNC Symbol;Acc:HGNC:84] | 9177 | -0.246 | -0.0577 | No |
| 124 | ADORA2B | adenosine A2b receptor [Source:HGNC Symbol;Acc:HGNC:264] | 9255 | -0.252 | -0.0594 | No |
| 125 | PANK3 | pantothenate kinase 3 [Source:HGNC Symbol;Acc:HGNC:19365] | 9286 | -0.255 | -0.0573 | No |
| 126 | GUCY1A1 | guanylate cyclase 1 soluble subunit alpha 1 [Source:HGNC Symbol;Acc:HGNC:4685] | 9420 | -0.266 | -0.0632 | No |
| 127 | PAICS | phosphoribosylaminoimidazole carboxylase and phosphoribosylaminoimidazolesuccinocarboxamide synthase [Source:HGNC Symbol;Acc:HGNC:8587] | 9475 | -0.272 | -0.0627 | No |
| 128 | SLC26A2 | solute carrier family 26 member 2 [Source:HGNC Symbol;Acc:HGNC:10994] | 9523 | -0.276 | -0.0616 | No |
| 129 | AMPD2 | adenosine monophosphate deaminase 2 [Source:HGNC Symbol;Acc:HGNC:469] | 9530 | -0.276 | -0.0573 | No |
| 130 | ENO1 | enolase 1 [Source:HGNC Symbol;Acc:HGNC:3350] | 9606 | -0.284 | -0.0583 | No |
| 131 | NPR1 | natriuretic peptide receptor 1 [Source:HGNC Symbol;Acc:HGNC:7943] | 9634 | -0.286 | -0.0554 | No |
| 132 | PNP | purine nucleoside phosphorylase [Source:HGNC Symbol;Acc:HGNC:7892] | 9657 | -0.288 | -0.0522 | No |
| 133 | AK4 | adenylate kinase 4 [Source:HGNC Symbol;Acc:HGNC:363] | 9682 | -0.290 | -0.0490 | No |
| 134 | ADCY2 | adenylate cyclase 2 [Source:HGNC Symbol;Acc:HGNC:233] | 9715 | -0.293 | -0.0465 | No |
| 135 | SHMT1 | serine hydroxymethyltransferase 1 [Source:HGNC Symbol;Acc:HGNC:10850] | 9727 | -0.294 | -0.0422 | No |
| 136 | GART | "phosphoribosylglycinamide formyltransferase, phosphoribosylglycinamide synthetase, phosphoribosylaminoimidazole synthetase [Source:HGNC Symbol;Acc:HGNC:4163]" | 9861 | -0.307 | -0.0474 | No |
| 137 | NOS3 | nitric oxide synthase 3 [Source:HGNC Symbol;Acc:HGNC:7876] | 9980 | -0.319 | -0.0511 | No |
| 138 | PPT2 | palmitoyl-protein thioesterase 2 [Source:HGNC Symbol;Acc:HGNC:9326] | 10103 | -0.331 | -0.0550 | No |
| 139 | PKLR | pyruvate kinase L/R [Source:HGNC Symbol;Acc:HGNC:9020] | 10120 | -0.333 | -0.0505 | No |
| 140 | GUCA2A | guanylate cyclase activator 2A [Source:HGNC Symbol;Acc:HGNC:4682] | 10138 | -0.335 | -0.0460 | No |
| 141 | MTHFD1 | "methylenetetrahydrofolate dehydrogenase, cyclohydrolase and formyltetrahydrofolate synthetase 1 [Source:HGNC Symbol;Acc:HGNC:7432]" | 10249 | -0.346 | -0.0486 | No |
| 142 | AMD1 | adenosylmethionine decarboxylase 1 [Source:HGNC Symbol;Acc:HGNC:457] | 10415 | -0.363 | -0.0553 | No |
| 143 | TAFAZZIN | "tafazzin, phospholipid-lysophospholipid transacylase [Source:HGNC Symbol;Acc:HGNC:11577]" | 10429 | -0.364 | -0.0500 | No |
| 144 | PPARGC1A | PPARG coactivator 1 alpha [Source:HGNC Symbol;Acc:HGNC:9237] | 10504 | -0.374 | -0.0494 | No |
| 145 | ACSL1 | acyl-CoA synthetase long chain family member 1 [Source:HGNC Symbol;Acc:HGNC:3569] | 10705 | -0.394 | -0.0583 | No |
| 146 | GUCA2B | guanylate cyclase activator 2B [Source:HGNC Symbol;Acc:HGNC:4683] | 10714 | -0.395 | -0.0521 | No |
| 147 | TMSB4X | thymosin beta 4 X-linked [Source:HGNC Symbol;Acc:HGNC:11881] | 10741 | -0.398 | -0.0472 | No |
| 148 | PFAS | phosphoribosylformylglycinamidine synthase [Source:HGNC Symbol;Acc:HGNC:8863] | 10943 | -0.424 | -0.0557 | No |
| 149 | ACACB | acetyl-CoA carboxylase beta [Source:HGNC Symbol;Acc:HGNC:85] | 10955 | -0.425 | -0.0492 | No |
| 150 | PDZD3 | PDZ domain containing 3 [Source:HGNC Symbol;Acc:HGNC:19891] | 10976 | -0.427 | -0.0433 | No |
| 151 | IMPDH1 | inosine monophosphate dehydrogenase 1 [Source:HGNC Symbol;Acc:HGNC:6052] | 11023 | -0.433 | -0.0395 | No |
| 152 | PPARA | peroxisome proliferator activated receptor alpha [Source:HGNC Symbol;Acc:HGNC:9232] | 11110 | -0.445 | -0.0385 | No |
| 153 | ELOVL5 | ELOVL fatty acid elongase 5 [Source:HGNC Symbol;Acc:HGNC:21308] | 11272 | -0.467 | -0.0431 | No |
| 154 | ACSL4 | acyl-CoA synthetase long chain family member 4 [Source:HGNC Symbol;Acc:HGNC:3571] | 11327 | -0.475 | -0.0391 | No |
| 155 | PDK4 | pyruvate dehydrogenase kinase 4 [Source:HGNC Symbol;Acc:HGNC:8812] | 11384 | -0.483 | -0.0351 | No |
| 156 | SLC25A16 | solute carrier family 25 member 16 [Source:HGNC Symbol;Acc:HGNC:10986] | 11411 | -0.488 | -0.0287 | No |
| 157 | NPPB | natriuretic peptide B [Source:HGNC Symbol;Acc:HGNC:7940] | 11463 | -0.497 | -0.0241 | No |
| 158 | VPS9D1 | VPS9 domain containing 1 [Source:HGNC Symbol;Acc:HGNC:13526] | 11495 | -0.501 | -0.0179 | No |
| 159 | PPAT | phosphoribosyl pyrophosphate amidotransferase [Source:HGNC Symbol;Acc:HGNC:9238] | 11534 | -0.505 | -0.0121 | No |
| 160 | DCAKD | dephospho-CoA kinase domain containing [Source:HGNC Symbol;Acc:HGNC:26238] | 11650 | -0.525 | -0.0121 | No |
| 161 | NPPA | natriuretic peptide A [Source:HGNC Symbol;Acc:HGNC:7939] | 11653 | -0.525 | -0.0031 | No |
| 162 | GUCA1B | guanylate cyclase activator 1B [Source:HGNC Symbol;Acc:HGNC:4679] | 12002 | -0.593 | -0.0202 | No |
| 163 | ADCY6 | adenylate cyclase 6 [Source:HGNC Symbol;Acc:HGNC:237] | 12005 | -0.594 | -0.0101 | No |
| 164 | RCVRN | recoverin [Source:HGNC Symbol;Acc:HGNC:9937] | 12169 | -0.636 | -0.0119 | No |
| 165 | NOS2 | nitric oxide synthase 2 [Source:HGNC Symbol;Acc:HGNC:7873] | 12262 | -0.665 | -0.0076 | No |
| 166 | ADSS2 | adenylosuccinate synthase 2 [Source:HGNC Symbol;Acc:HGNC:292] | 12376 | -0.701 | -0.0043 | No |
| 167 | DIP2A | disco interacting protein 2 homolog A [Source:HGNC Symbol;Acc:HGNC:17217] | 12433 | -0.719 | 0.0037 | No |
| 168 | HPCA | hippocalcin [Source:HGNC Symbol;Acc:HGNC:5144] | 12763 | -0.924 | -0.0061 | No |
| 169 | MYC | "MYC proto-oncogene, bHLH transcription factor [Source:HGNC Symbol;Acc:HGNC:7553]" | 12801 | -0.970 | 0.0078 | No |
Table: GSEA details [plain text format]

  

Fig 2: GOBP\_PURINE\_CONTAINING\_COMPOUND\_BIOSYNTHETIC\_PROCESS      
 Blue-Pink O' Gram in the Space of the Analyzed GeneSet

  

Fig 3: GOBP\_PURINE\_CONTAINING\_COMPOUND\_BIOSYNTHETIC\_PROCESS: Random ES distribution      
 Gene set null distribution of ES for **GOBP\_PURINE\_CONTAINING\_COMPOUND\_BIOSYNTHETIC\_PROCESS**
